# Scoring sleep with artificial intelligence enables quantification of sleep stage ambiguity: hypnodensity based on multiple expert scorers and auto-scoring

**DOI:** 10.1093/sleep/zsac154

**Published:** 2022-07-03

**Authors:** Jessie P Bakker, Marco Ross, Andreas Cerny, Ray Vasko, Edmund Shaw, Samuel Kuna, Ulysses J Magalang, Naresh M Punjabi, Peter Anderer

**Affiliations:** Philips Sleep and Respiratory Care, Pittsburgh, PA,USA; Philips Sleep and Respiratory Care, Vienna, Austria; Philips Sleep and Respiratory Care, Vienna, Austria; Philips Sleep and Respiratory Care, Pittsburgh, PA,USA; Philips Sleep and Respiratory Care, Pittsburgh, PA,USA; Perelman School of Medicine, University of Pennsylvania, Philadelphia, PA,USA; Corporal Michael J. Crescenz Veterans Affairs Medical Center, Philadelphia, PA,USA; Division of Pulmonary, Critical Care, and Sleep Medicine, Ohio State University Wexner Medical Center, Columbus, OH, USA; Division of Pulmonary, Critical Care, and Sleep Medicine, University of Miami, Miami FL, USA; Philips Sleep and Respiratory Care, Vienna, Austria

**Keywords:** sleep stages, polysomnography, artificial intelligence, machine learning, validation, hypnodensity

## Abstract

**Study Objectives:**

To quantify the amount of sleep stage ambiguity across expert scorers and to validate a new auto-scoring platform against sleep staging performed by multiple scorers.

**Methods:**

We applied a new auto-scoring system to three datasets containing 95 PSGs scored by 6–12 scorers, to compare sleep stage probabilities (hypnodensity; i.e. the probability of each sleep stage being assigned to a given epoch) as the primary output, as well as a single sleep stage per epoch assigned by hierarchical majority rule.

**Results:**

The percentage of epochs with 100% agreement across scorers was 46 ± 9%, 38 ± 10% and 32 ± 9% for the datasets with 6, 9, and 12 scorers, respectively. The mean intra-class correlation coefficient between sleep stage probabilities from auto- and manual-scoring was 0.91, representing excellent reliability. Within each dataset, agreement between auto-scoring and consensus manual-scoring was significantly higher than agreement between manual-scoring and consensus manual-scoring (0.78 vs. 0.69; 0.74 vs. 0.67; and 0.75 vs. 0.67; all *p* < 0.01).

**Conclusions:**

Analysis of scoring performed by multiple scorers reveals that sleep stage ambiguity is the rule rather than the exception. Probabilities of the sleep stages determined by artificial intelligence auto-scoring provide an excellent estimate of this ambiguity. Compared to consensus manual-scoring, sleep staging derived from auto-scoring is for each individual PSG noninferior to manual-scoring meaning that auto-scoring output is ready for interpretation without the need for manual adjustment.

**Statement of Significance**

The common belief that the majority of polysomnography epochs are staged unequivocally by expert scorers is rejected using three independent datasets containing 95 PSGs scored by 6–12 scorers. Our analyses demonstrate sleep staging ambiguity in 70%–80% of epochs; consequently, assigning a single sleep stage to each epoch and presenting sleep architecture as a hypnogram is misleading. A hypnodensity chart representing sleep stage probabilities reflects this sleep staging ambiguity while providing all of the information contained in a hypnogram. We present validation data demonstrating excellent reliability of sleep stage probabilities derived from an auto-scoring platform vs. multiple expert scorers, and recommend that traditional assignment of a single sleep stage per epoch (hypnogram) be replaced by sleep stage probabilities (hypnodensity).

## Introduction

Sleep staging is typically undertaken manually according to published criteria based on electroencephalogram (EEG), electrooculogram (EOG) and electromyogram (EMG) signals collected during polysomnography (PSG). Until 2007, the only widespread standard for sleep staging was that of Rechtschaffen and Kales (R&K) [1]; subsequently, the *Manual for the Scoring of Sleep and* Associated Events was published by the American Academy of Sleep Medicine (AASM) [2] with the goal of simplifying the scoring criteria for easier implementation [3]. In 2009, Danker-Hopfe et al. [4] reported greater inter-scorer agreement when following the AASM criteria compared to R&K (Cohen’s kappa values 0.76 and 0.68, respectively). Since then, several studies have reported inter-scorer reliability data for sleep staging according to AASM criteria, with kappa values ranging from 0.63 to 0.76 [4–7]. Manual staging of NREM sleep into its three stages has been found to be unreliable in clinical practice when it is performed by one scorer [8]. As stated in the paper describing the 2007 AASM task force, “No visual-based scoring system will ever be perfect, as all methods are limited by the physiology of the human eye and visual cortex, individual differences in scoring experience, and the ability to detect events viewed using a 30-s epoch” [3].

Numerous attempts have been made to develop and validate automated sleep staging systems according to both R&K [9–16] and AASM criteria [8, 17–27]. With recent advances in machine learning, powerful methods are now available for sleep stage classification leading to auto-scoring systems with a performance comparable to manual-scoring. Auto-scoring offers several potential advantages to manual-scoring: (1) increased efficiency resulting from the avoidance of manual data processing; (2) attainment of reproducible sleep staging, particularly for NREM sub-classification; and (3) the ability to glean additional insights regarding the ambiguity of scoring a given epoch. The concept of sleep stage probabilities, that is, the probability of each sleep stage being assigned to a given epoch, has been referred to as hypnodensity and visualized previously [23].

We evaluated a new sleep staging system which allows the user to interpret sleep stage probabilities displayed as hypnodensity graph. Rather than validation against a single scorer, a more robust approach for assessing the performance of an auto-scoring algorithm is to compare the output against multiple scorers. For the present study, we accessed data from three prior multi-scorer studies [6, 18, 19] to compare sleep stage probabilities generated from the algorithm against sleep stage probabilities derived from multiple scorers. Our over-arching objectives were to determine sleep staging variability across multiple scorers, to describe the concept of sleep stage probabilities as a means of assessing the degree of ambiguity of scoring, and to compare the performance of the auto-scoring algorithm against manual scorers.

## Methods

We accessed de-identified data collected in three previous studies, each described below. None of the PSGs reported in the current study were used for algorithm training. Approval for these analyses was obtained from the Western Institutional Review Board (Protocols 20192184, 20192293, 20192296). No new data were generated in support of this research. The datasets were collected during studies that were not funded by the National Institutes of Health; as such, there was no data sharing policy in place at the time the data were collected. It may, however, be possible to make the datasets available to other parties by contacting the corresponding author.

### Algorithm training

Algorithm training was undertaken separately from validation using the SIESTA dataset. The dataset consists of 394 PSGs from healthy adults (two PSGs each in 98 males and 99 females aged 20–95 years with approximately the same number of males and females per decade) and 194 PSGs from adults with sleep-disordered breathing, insomnia related to generalized anxiety disorder as well as mood disorders, periodic limb movement disorders and Parkinson’s disease [28]. 516 of the 588 PSGs were scored by three experts according to R&K criteria, 60 PSGs were scored by three experts according to R&K and by two experts according to AASM criteria, and 12 PSGs were scored by three experts according to R&K and by six experts according to AASM criteria. Scorers were from a pool of thirty experts from eight different sleep labs which enabled us the modeling of sleep stage ambiguity. The entire SIESTA data, including more than 1 800 000 manually scored sleep stages, was used for training the auto-scoring algorithm. Details on algorithm input signals and classifier architecture can be found in the on-line supplement.

### Algorithm validation

#### Datasets

Dataset A consisted of 70 PSGs scored by six technologists representing five sites [18, 29], from which we analyzed all currently available data (69 PSGs with six scorings and one PSG with five scorings). The PSGs (Compumedics; Abbotsville, Victoria Australia) were collected during a study examining the impact of SDB amongst women aged 40–57 years. Dataset B consisted of 15 PSGs scored by nine technologists representing nine sites [6]. The PSGs (Embla Systems; Ontario, Canada) were accessed from a single clinic, identified to ensure a wide range of SDB severity. Dataset C consisted of ten PSGs scored by 12 technologists representing 6 sites [19], which were initially scored by 4 technologists representing 4 sites, and subsequently scored by an additional 8 technologists representing 2 sites. The PSGs (Alice; Philips; PA, USA) were originally accessed from three clinics, identified to ensure an approximately even mix of referral reasons across sites. The raw data (European Data Format;.edf) were imported into Sleepware G3 Version 4.0.0 (Philips; PA, USA). The Somnolyzer auto-scoring algorithm was used to generate a sleep stage probability (hypnodensity; described further below) for each individual epoch, as well as traditional sleep staging (hypnogram). No manual editing was undertaken after the auto-scoring algorithm was deployed.

### Statistical analyses

Statistical analyses were performed using Matlab (Version 2019b; MathWorks, Inc., Natick, MA). To assess sleep staging ambiguity, we evaluated the complete agreement across manual scorers, varying the number of scorers from two up to the total number of scorers available. Agreement was determined as the average of the agreements between all possible permutations of the sub-groups. To assess agreement between sleep stage probabilities (continuous variables) derived from manual- and auto-scoring, intra-class correlation coefficients (ICCs) for absolute agreement were calculated (ICC (2,1) according to [30]) and compared against published thresholds [31]. To assess the agreement between sleep stages based on manual- and auto-scoring (categorical variables), Cohen’s kappa statistics [32] were calculated comparing auto-scoring as well as each individual scorer with: (1) each remaining scorer; (2) the consensus of the remaining scorers; and (3) any of the remaining scorers; that is, agreement was counted as long as the test-scorer agreed with at least one of the remaining scorers. The unbiased consensus for each scorer was the majority vote of the remaining scorers. In the case of ties, scorers with a higher consensus with the group (as measured by Cohen’s kappa) were considered more reliable and thus their assessments were weighted heavier than the other scorers [23]. Kappa values were compared with paired-samples *t*-tests.

## Results

Descriptive data and PSG parameters averaged across scorers are provided in Table 1. Dataset A was generated during a study examining the impact of SDB amongst women aged 40–57 years. The BMI in this group was 32. 9 ± 9.2 kg/m², which is within the reported range for U.S. woman aged 40–59 years [33]. Dataset B consisted of patients selected from a single clinic, identified to ensure a wide range of SDB severity based on AHI. The higher BMI in Dataset B (37.7 ± 12.0 kg/m²) can therefore be explained by the the higher AHI in Dataset B (22.5 ± 17.1 and 7.4 ± 12.2 events/hour for Datasets B and A, respectively) [34]. The rather low total sleep times (on average between 5.9 and 6.6 hours/night across Datasets A–C) are probably explained by both the first-night effect and the severity of sleep-disordered breathing [35].

**Table 1.** Descriptive data for the three validation datasets

	Dataset A	Dataset B	Dataset C
Number of PSGs, *n*	70	15	10
Number of scorer,; *n*	6*	9	12
Females/women, *n* (%)	70 (100%)	3 (20%)	3 (30%)
Age (years)	51.1 ± 4.2	46.5 ± 12.0	57.9 ± 16.6
Body mass index (kg/m²)	32.9 ± 9.2	39.7 ± 19.0	[Not collected]
Time in bed (h)	7.9 ± 1.0	6.9 ± 0.7	7.9 ± 1.0
Total sleep time (h)	6.6 ± 0.9	5.9 ± 0.8	6.0 ± 0.8
Sleep efficiency (%)	84.0 ± 7.6	85.7 ± 7.2	76.2 ± 8.7
Time in N1 (min)	42.8 ± 17.5	61.5 ± 19.7	77.2 ± 39.4
Time in N2 (min)	244.4 ± 45.7	200.3 ± 34.4	191.1 ± 59.9
Time in N3 (min)	30.4 ± 21.3	43.9 ± 31.3	32.1 ± 23.9
Time in REM (min)	80.9 ± 24.6	50.6 ± 24.8	57.8 ± 23.8
Arousal Index (events/h)	16.9 ± 6.9	30.1 ± 15.5	23.9 ± 10.8
AHI (events/h)	7.4 ± 12.3	24.7 ± 18.2	32.4 ± 22.1
ODI (events/h)	7.3 ± 12.2	22.5 ± 17.1	26.8 ± 19.3

All data are presented as mean ± SD unless otherwise indicated.

*For Dataset A, scoring from six technologists was available for 69 PSGs, while scoring from five technologists was available for one PSG.

### Sleep staging agreement across scorers

**Figure 1.**
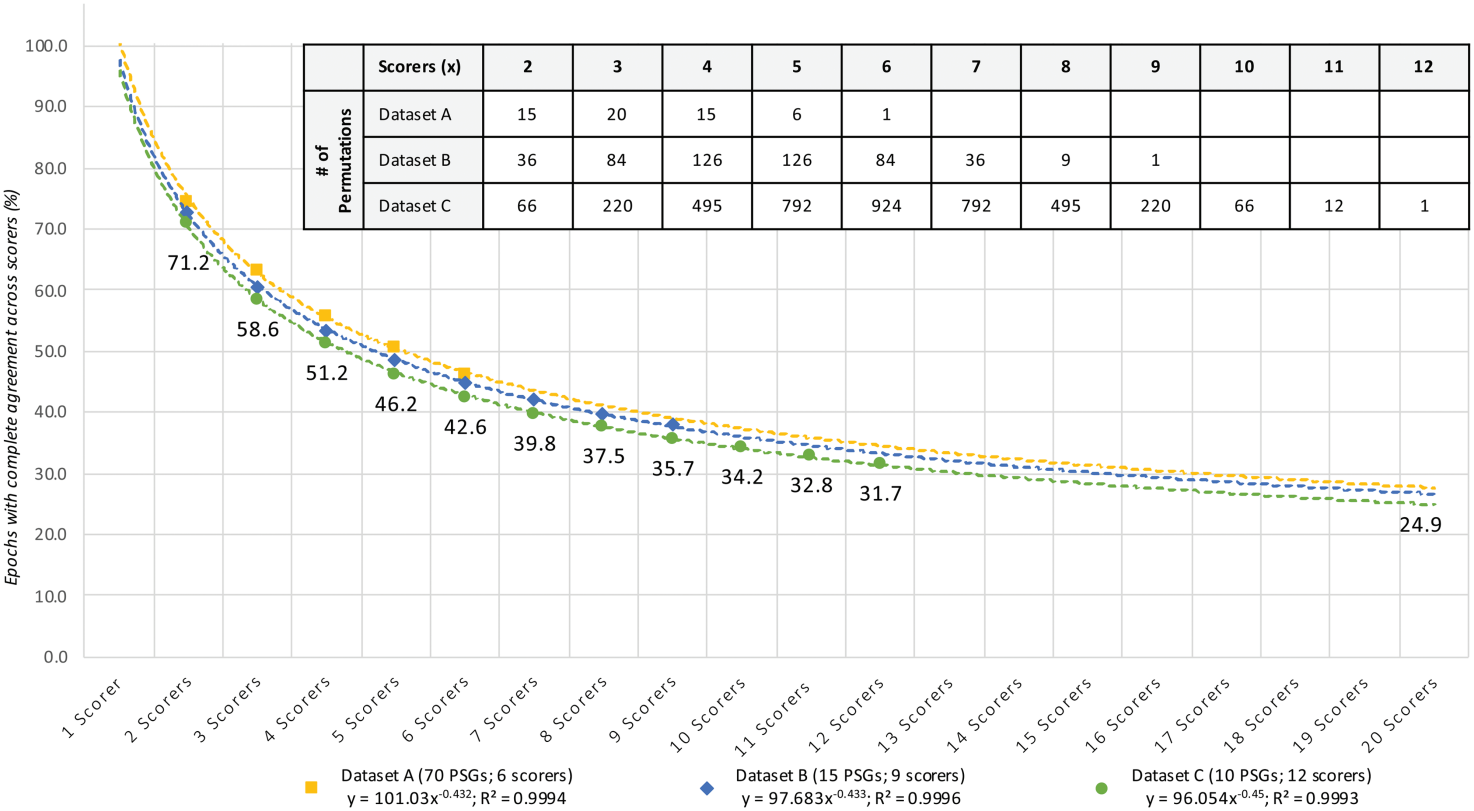
Percentage of all epochs with complete sleep staging agreement across multiple scorers in each dataset. The number of scorers compared is shown on the *x*-axis; the percentage of complete agreement across the compared scorers is shown on the *y-*axis. The mean of all possible permutations for choosing *x* scorers out of the available scorers for Dataset A is shown in yellow; Dataset B is shown in blue; Dataset C is shown in green (observed values; filled markers). The table in the upper right corner describes the number of permutations for each combination of scorers in each dataset. The values shown in the figure depict the observed percentage of epochs with complete agreement for Dataset C, which had the highest number of scorers. The reduction in complete agreement alongside the increasing number of scorers follows an almost-perfect power function (dashed lines for each dataset). In addition to the coefficients and exponents, the explained variances (*R*^2^) are shown. Modeled together, the power function *y* = *ax*^*b*^ has a coefficient *a*_MEAN_ of 98 and an exponent *b*_MEAN_ of ‐0.44.

The mean percentage of epochs per PSG with complete agreement across all 6, 9, or 12 scorers was 46.4 ± 8.6%, 37.9 ± 10.3%, and 31.7 ± 9.4% for Datasets A–C, respectively. Figure 1 shows the decreasing agreement alongside an increasing number of scorers. Within each dataset, the epochs from all PSGs were pooled, and the percentage of epochs for which there was complete agreement across scorers was calculated for all possible permutations of two scorers, three scorers, and so on up to the maximum number of scorers per dataset. The decline in scoring agreement followed a power function *y* = *ax*^*b*^ (shown as a dotted line for each dataset) where *y* is the percentage of epochs with complete agreement, *x* is the number of scorers, *a* is the coefficient, and *b* is the exponent. This model explained almost 100% of the variance in all three datasets (all *R*^2^ > 0.999). The power functions are very similar for each dataset, indicating a robust effect independent of the dataset and the scorers. The observed values, which indicate complete agreement between two scorers, was attained for less than 75% of the epochs in all three datasets, and dropped below 50% when five or more scorers were compared. The model averaged across all three datasets [*y* = 98(*x*^‐0.44^)] predicts that fewer than 25% of epochs will be agreed upon by 24 or more scorers, indicating that the vast majority of sleep epochs can be considered ambiguous. Supplementary Figure S1 presents these results for each sleep stage separately. For epochs scored as N2, the decline in agreement with increasing numbers of manual scorers was similar to the decline observed for all stages while for epochs scored as W and R the decline was weaker and for epochs scored as N3 and N1 the decline was stronger.

### Agreement between sleep stage probabilities derived from manual- and auto-scoring

**Figure 2.**
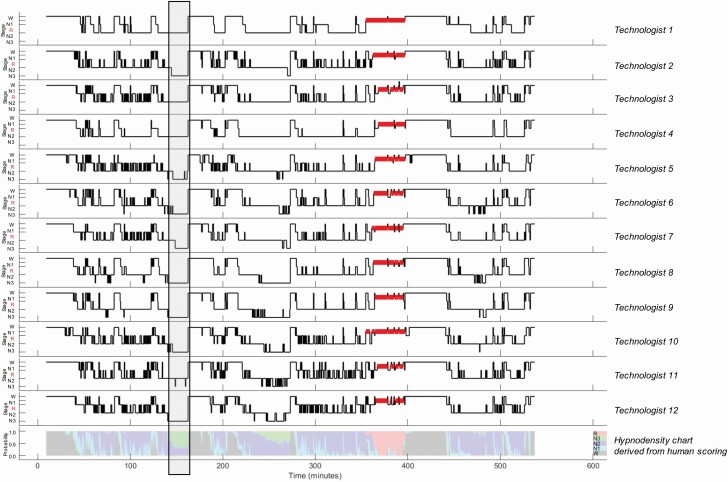
A representative example of 12 manually scored hypnograms and the derived hypnodensity chart for a single PSG. This PSG, drawn from Dataset C, was scored by 12 technologists. Individual hypnograms are presented in panels 1–12. Panel 13 depicts the hypnodensity chart, based on sleep stage probabilities derived from the 12 scorers. Color codes for the hypnodensity chart are shown in the legend (wake [W]: gray; N1: cyan; N2: blue; N3: green; REM [R]: red). The period of time depicted in the shaded box is described further in the text.

Given this level of variability across scorers, presenting sleep staging as a hypnogram in which each epoch is assigned a single “true” sleep stage may be misleading. In contrast, a hypnodensity chart reflects sleep stage ambiguity while providing all of the information contained in a hypnogram. A representative example is provided in Figure 2, in which manually scored hypnograms for a single PSG are shown in panels 1–12. Panel 13 depicts a hypnodensity chart showing sleep stage probabilities for each epoch. At any given timepoint along the *x*-axis in the hypnodensity chart, if the entirety of the *y*-axis is represented by a single color, then there is 100% agreement across scorers that the epoch in question reflects the sleep stage represented by that color. Conversely, a time-point on the *x*-axis that is associated with multiple colors across the *y*-axis reflects greater disagreement across scorers. For example, within the period of time shown in the shaded box of Figure 2, 6 of the 12 scorers identified a mostly consolidated block of N3; however, scorers 1, 3, 4, and 11 identified a mostly consolidated block of N2 while scorers 5 and 7 scored a combination of N2 and N3. As 6 of the 12 scorers were in agreement, applying a majority rule would result in this period being staged as N3; however, the hypnodensity chart in panel 13 shows a 0.6 probability of N3 with a 0.4 probability of N2 during this period.

**Figure 3.**
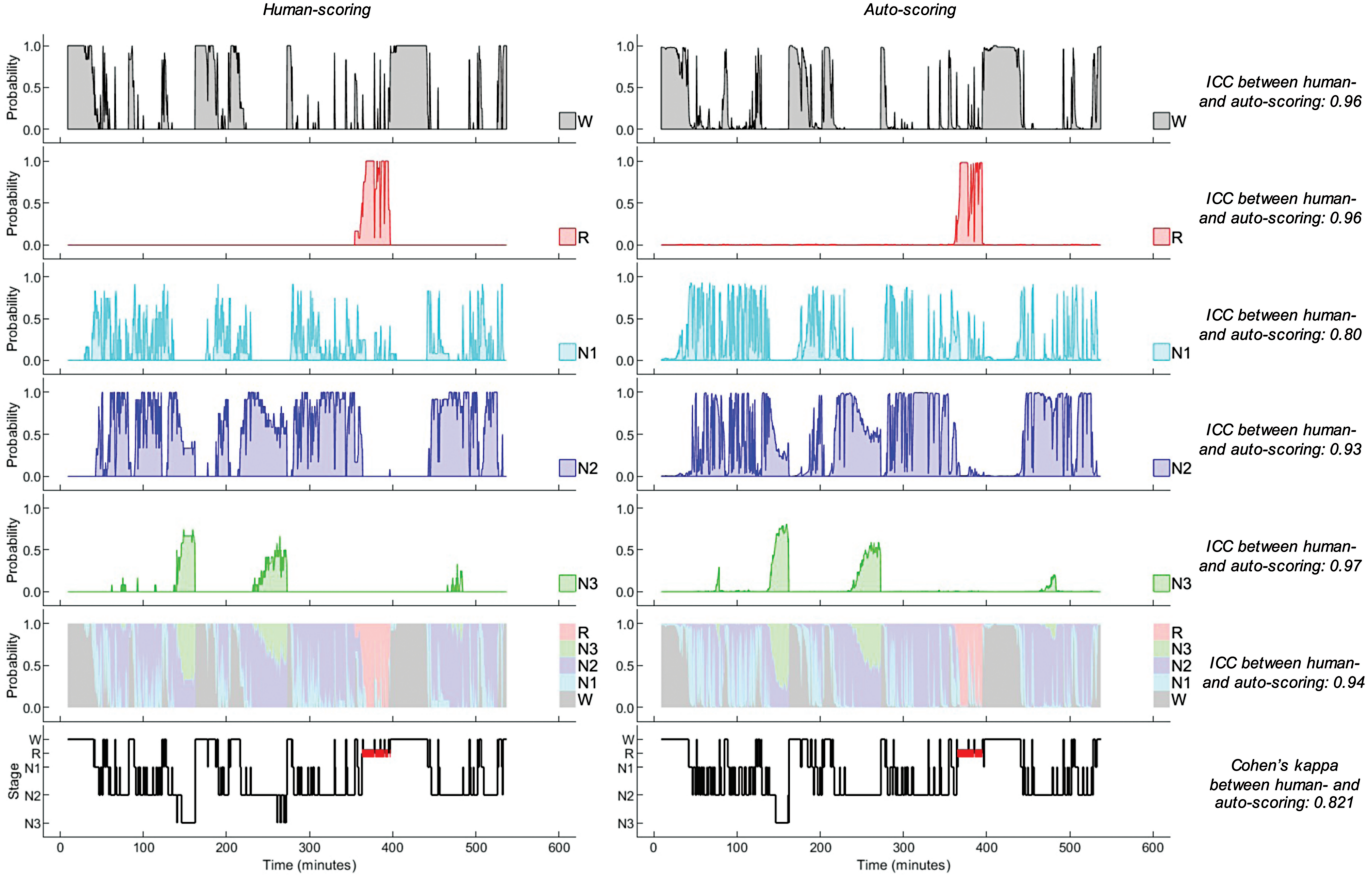
A representative example of hypnodensity charts derived from manual- and auto-scoring for a single PSG. Sleep stage probabilities for individual sleep stages (panels 1–5) derived from the 12 scorers (left) and Somnolyzer auto-scoring (right). The PSG depicted in this figure is the same as the PSG described in Figure 2. The probability distributions were compared quantitatively with an ICC value as shown. Panel 6 shows the hypnodensity charts that combine the individual sleep stage probabilities into a stacked area graph for manual- (left) and auto-scoring (right), also compared quantitatively with an ICC value. Color codes for the hypnodensity chart are shown in the legend (wake [W]: gray; N1: cyan; N2: blue; N3: green; REM [R]: red). Finally, panel 7 shows a hypnogram derived from majority vote of manual- (left) and auto-scoring (right), which can be compared quantitatively with a Cohen’s kappa value as shown. ICC values of 0.75–0.90 and ≥0.90 represent good and excellent agreement, respectively [31]. A kappa value above 0.80 represents almost-perfect agreement [32].

The hypnodensity chart depicted in Figure 2 was made possible by having sleep staging performed by multiple scorers, which is rarely available in clinical settings. As the output of the Somnolyzer algorithm is sleep stage probabilities per epoch, it is possible to create a hypnodensity chart from auto-scoring which is directly comparable to sleep stage probabilities calculated across multiple scorers. Figure 3 depicts sleep stage probabilities for the same PSG shown in Figure 2, with probabilities derived from manual-scoring on the left and Somnolyzer on the right. Panels 1–5 show the probabilities of each sleep stage individually, while panel 6 is a hypnodensity chart which merges panels 1–5 into a single figure. The hypnograms in panel 7 represent the majority vote derived from manual scorers (left) and for the final sleep stage assigned by auto-scoring (right). The similarities between individual sleep stage probabilities from manual-scoring (panels 1–5; left) vs. Somnolyzer (panels 1–5; right) for this PSG are reflected quantitatively by ICC values ranging from 0.80 (N1) to 0.97 (N3). The ICC values can be interpreted as the extent to which the agreement across scorers can be predicted by the auto-scoring’s sleep stage probabilities. When panels 1–5 were combined to form a hypnodensity chart (panel 6), the overall ICC value was 0.94, representing excellent agreement between manually and auto-scored sleep stage probabilities [31]. The hypnograms in panel 7 assign a single sleep stage per epoch; the resulting Cohen’s kappa of 0.821 represents almost-perfect agreement between manual- and auto-scored sleep staging for this PSG [32].

**Figure 4.**
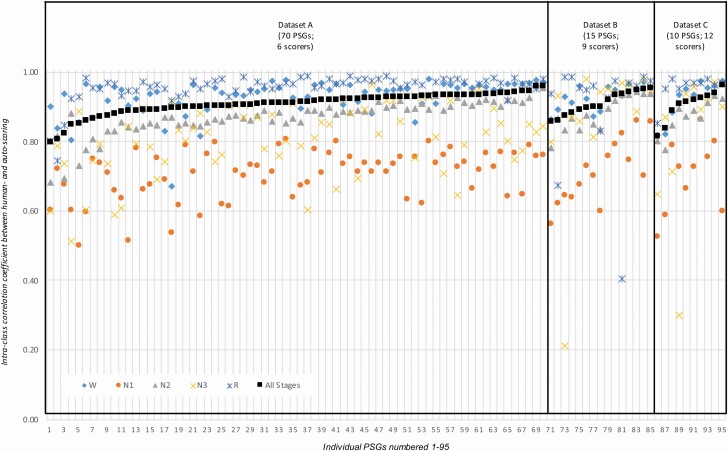
Agreement between sleep stage probabilities derived from manual- and auto-scoring for each individual PSG. ICC values for manual- vs. auto-scored sleep stage probabilities for all 95 individual PSGs, broken down by individual sleep stages (colored markers) and all five stages (black squares). Note that the PSGs are sorted by ascending ICC values for the five-stage comparison within each dataset.

Table 2 contains the ICC values comparing manual- vs. auto-scored sleep stage probabilities averaged across all PSGs in each dataset. The mean ICC value for all sleep stage discriminations was identical (0.91) in Datasets A–C; for stages W and R, the correlations were even higher (≥0.93 and 0.96, respectively). Concerning NREM subclassification, the correlations were 0.88–0.89 for N2 and 0.85–0.94 for N3, while the correlations for N1 are lower (0.72–0.74); however, the correlations for individual sleep stages were again consistent across the three datasets. In addition to the ICCs aggregated across all PSGs in Table 2, Figure 4 displays an individual ICC value for each PSG. The ICCs for the five-stage comparison were above 0.8 for all 95 PSGs, while for 70 PSGs (74%) the ICC value exceeded 0.9 indicating excellent reliability [31]. As expected from the data in Table 2, the ICCs between manual- and auto-scoring of W (blue diamonds) and R (blue asterixes) were slightly higher than the ICCs for all stages (black squares), and the individual ICCs for N2 (gray triangles) are slightly lower. While the ICCs for N1 (orange circles) typically show the lowest values, there is no single PSG with an ICC between manual- and auto-scoring that is below 0.5 (indicating poor reliability [31]) for N1. The only individual ICCs below 0.5 are from two PSGs from Dataset B and one PSG from Dataset C; these PSGs either had very little manually scored N3 (<5 min; see Supplementary Figures S2, S3, S6, and S7) or R (<2 min; see Supplementary Figures S4 and S5), and thus these ICC values are not statistically meaningful.

**Table 2.** Intra-class correlation coefficients between sleep stage probabilities derived from manual- and auto-scoring

	Mean ICC (95% CI)					
	All stages	W	N1	N2	N3	R
Dataset A	0.91 (0.910–0.911)	0.94 (0.942–0.944)	0.72 (0.711–0.730)	0.88 (0.843–0.902)	0.85 (0.821–0.865)	0.96 (0.959–0.960)
Dataset B	0.91 (0.912–0.915)	0.94 (0.934–0.940)	0.73 (0.711–0.747)	0.89 (0.880–0.902)	0.94 (0.940–0.948)	0.96 (0.954–0.960)
Dataset C	0.91 (0.907–0.910)	0.93 (0.920–0.932)	0.74 (0.727–0.755)	0.89 (0.881–0.890)	0.92 (0.911–0.920)	0.97 (0.962–0.970)

ICCs were calculated for absolute agreement (ICC [2,1]).30 ICC values of 0.5–0.75, 0.75–0.90 and ≥0.90 represent moderate, good, and excellent agreement, respectively

^[31]^.

### Agreement between sleep stages from manual- and auto-scoring

In Table 3, we present Cohen’s kappa statistics for the five-stage comparison as well as for each of the five individual sleep stages (W, N1, N2, N3, and R) aggregated for all PSGs within each dataset, comparing manual- and auto-scoring against three different comparators. First, auto-scoring and each individual scorer was compared pair-wise to each of the remaining scorers, and the resulting kappa values are averaged. Second, each individual scorer was compared to the consensus of the remaining scorers; to allow direct comparison between manual- and auto-scoring kappa values, auto-scoring was compared to the same unbiased consensus as each individual scorer, and the resulting kappa values were averaged. Third, each scorer was compared to all remaining scorers, where each epoch was considered correct if there was agreement with at least one other scorer; again, auto-scoring was compared to the same combinations of scorers, and the resulting kappa values were averaged.

**Table 3.** Cohen’s kappa values comparing manual- and auto-scoring against three different comparators

	All stages		W		N1		N2		N3		R	
	Manual	Auto	Manual	Auto	Manual	Auto	Manual	Auto	Manual	Auto	Manual	Auto
Dataset A												
vs. individual scorers	0.62 ± 0.071	0.69 ± 0.054	0.80 ± 0.053	0.83 ± 0.052	0.32 ± 0.119	0.39 ± 0.113	0.59 ± 0.110	0.67 ± 0.081	0.42 ± 0.156	0.56 ± 0.185	0.79 ± 0.041	0.84 ± 0.041
vs. unbiased consensus of scorers	0.69 ± 0.063	0.78 ± 0.013	0.85 ± 0.046	0.89 ± 0.004	0.42 ± 0.099	0.51 ± 0.025	0.68 ± 0.094	0.77 ± 0.017	0.51 ± 0.151	0.69 ± 0.058	0.83 ± 0.032	0.90 ± 0.007
vs. any scorer	0.90 ± 0.050	0.96 ± 0.005	0.93 ± 0.045	0.96 ± 0.001	0.80 ± 0.089	0.88 ± 0.008	0.90 ± 0.072	0.96 ± 0.006	0.89 ± 0.113	0.99 ± 0.013	0.93 ± 0.034	0.98 ± 0.002
Dataset B												
vs. individual scorers	0.62 ± 0.062	0.66 ± 0.033	0.76 ± 0.047	0.79 ± 0.030	0.33 ± 0.097	0.41 ± 0.095	0.60 ± 0.090	0.65 ± 0.048	0.65 ± 0.093	0.72 ± 0.071	0.80 ± 0.056	0.82 ± 0.040
vs. unbiased consensus of scorers	0.69 ± 0.038	0.75 ± 0.005	0.82 ± 0.042	0.85 ± 0.003	0.42 ± 0.053	0.53 ± 0.028	0.67 ± 0.057	0.74 ± 0.008	0.72 ± 0.065	0.81 ± 0.020	0.85 ± 0.047	0.87 ± 0.005
vs. any scorer	0.95 ± 0.028	0.97 ± 0.002	0.96 ± 0.021	0.98 ± 0.002	0.92 ± 0.036	0.95 ± 0.005	0.95 ± 0.036	0.98 ± 0.004	0.96 ± 0.033	1.00 ± 0.001	0.96 ± 0.039	0.97 ± 0.002
Dataset C												
vs. individual scorers	0.60 ± 0.055	0.64 ± 0.038	0.75 ± 0.049	0.76 ± 0.041	0.32 ± 0.086	0.39 ± 0.084	0.57 ± 0.068	0.62 ± 0.052	0.50 ± 0.177	0.55 ± 0.080	0.84 ± 0.050	0.88 ± 0.036
vs. unbiased consensus of scorers	0.69 ± 0.045	0.76 ± 0.004	0.82 ± 0.042	0.82 ± 0.006	0.44 ± 0.078	0.54 ± 0.012	0.66 ± 0.057	0.76 ± 0.006	0.59 ± 0.133	0.75 ± 0.005	0.88 ± 0.046	0.93 ± 0.003
vs. any scorer	0.96 ± 0.024	0.99 ± 0.001	0.97 ± 0.021	0.98 ± 0.002	0.94 ± 0.036	0.97 ± 0.002	0.96 ± 0.026	0.99 ± 0.001	0.96 ± 0.034	1.00 ± 0.001	0.97 ± 0.033	1.00 ± 0.001

Data are presented as mean ± SD Cohen’s kappa values. Individual Scorers: Pairwise comparison between the evaluated scorer (manual- or auto-scoring) and each remaining scorer (resulting kappa values are averaged). Unbiased Consensus of Scorers: Each evaluated scorer is compared to the consensus of the remaining scorers (unbiased consensus), and the auto-scoring is compared to the same unbiased consensus for each scorer (resulting kappa values are averaged). Any Scorer: Each evaluated scorer is compared to all remaining scorers, where each epoch is considered correct if at least one of the remaining scorers agreed with the evaluated scorer, and the auto-scoring is compared to the same combinations of scorers (resulting kappa values are averaged). Kappa values of 0.21–0.4, 0.41–0.6, 0.61–0.8, and >0.8 represent fair, moderate, substantial, and almost-perfect agreement

^[32]^.

As shown in Table 3, across all three datasets Somnolyzer outperformed manual-scoring for the five-stage comparison as well as for each individual stage, regardless of the comparator. Moreover, consistent with the results in Table 2, the highest kappa values were observed for W and R, while lower kappa values were observed for NREM stages, particularly N1. Finally, lower kappa values were observed with the “individual scorers” comparator due to the aforementioned inter-scorer variability, while highest kappa values were observed with the “any scorer” comparator. For example, the kappa values for Somnolyzer vs. all individual scorers for N3 ranged from 0.55 to 0.72; however, the kappa values for Somnolyzer vs. any scorer for N3 ranged from 0.99 to 1.00, meaning that when Somnolyzer scored an epoch as N3, in almost all cases at least one scorer also identified N3. Confusion matrices showing the number and percentages of epochs classified as stages W, N1, N2, N3, and R by auto-scoring vs. each comparator are provided in Supplementary Tables S1–S3.

**Figure 5.**
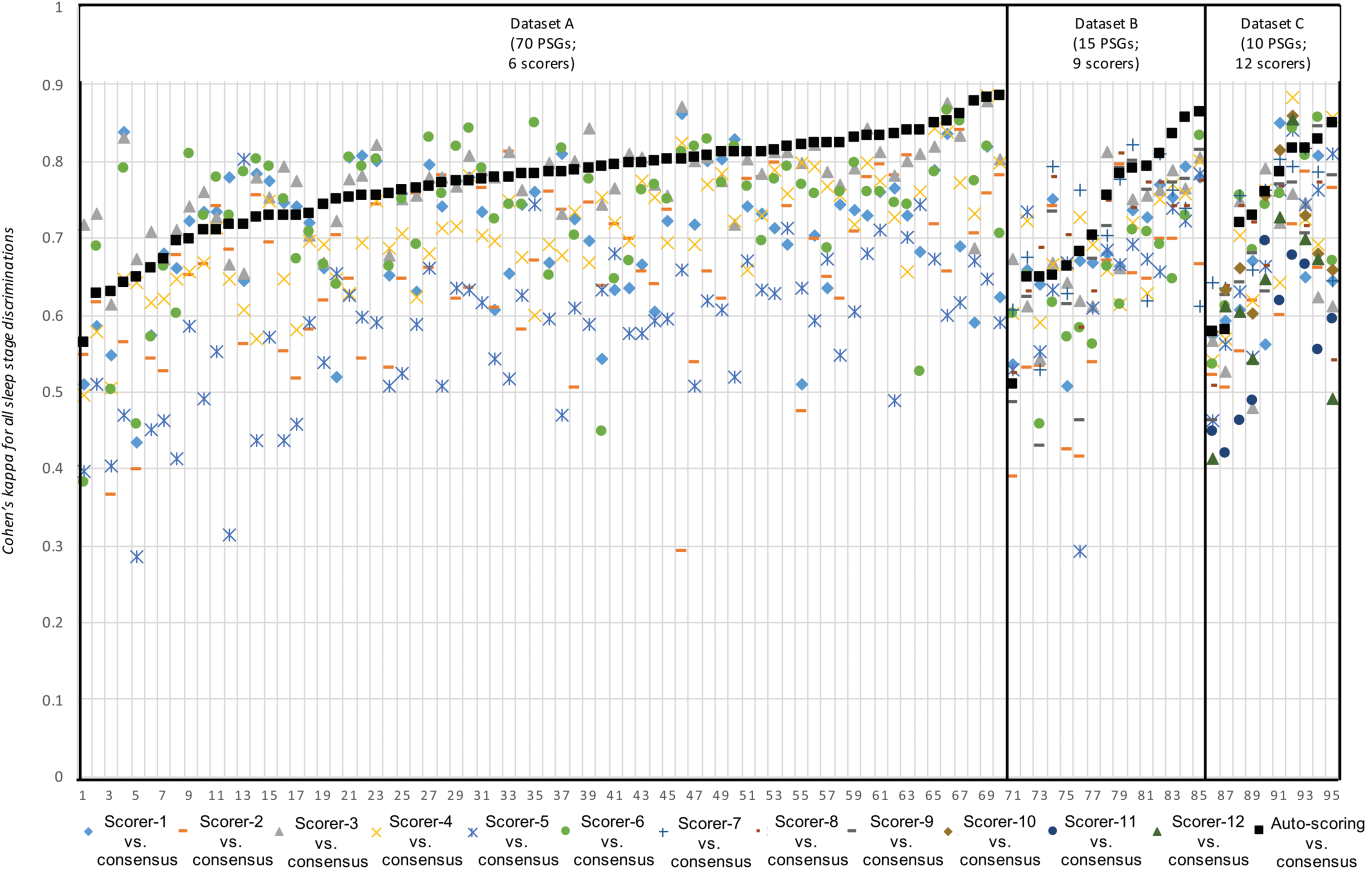
Agreement between sleep stage assignments derived from manual- and auto-scoring for each individual PSG. Cohen’s kappa values for manual- and auto-scored sleep staging vs. the unbiased consensus of manual-scoring for all 95 individual PSGs. The colored markers represent the kappa values between each individual scorer and the unbiased consensus (majority vote) of the remaining scorers. Black squares represent the averaged kappa values between auto-scoring and the unbiased consensus for each scorer. Note that the PSGs are sorted by ascending kappa values for auto-scoring vs. consensus manual-scoring within each dataset.

Figure 5 displays individual five-stage Cohen’s kappa values for each PSG. The colored markers represent the kappa values between each individual scorer and the unbiased consensus (majority vote) of the remaining scorers, while the black markers represent the averaged kappa values between auto-scoring and the same unbiased consensus. The kappa values based on auto-scoring ranged from 0.52 to 0.89. For 91 of the 95 PSGs (96%), the kappa value exceeded 0.6 indicating substantial agreement [32]. The kappa values based on manual-scoring ranged from 0.28 to 0.88. For each PSG, auto-scoring outperformed at least three scorers; in 54 PSGs (57%) auto-scoring outperformed all scorers. Compared to the kappa value averaged across scorers, the kappa value for auto-scoring was higher for 96% of the PSGs. As such, auto-scoring kappa values were statistically significantly higher than manual-scoring kappa values in all three datasets (Dataset A: auto-scoring 0.780 ± 0.066 vs. manual-scoring 0.691 ± 0.067, *p* = 1.2 × 10^‐24^; Dataset B: auto-scoring 0.741 ± 0.097 vs. manual-scoring 0.674 ± 0.109, *p* = 1.5 × 10^‐4^; Dataset C: auto-scoring 0.749 ± 0.095 vs. manual-scoring 0.673 ± 0.086, *p* = 1.4 × 10^‐3^).

Neither manual nor auto-scoring accuracy were affected by age. Partial correlation coefficients between age and Cohen’s kappa controlling for SDB severity as measured by the AHI were ‐0.12 (*p* = 0.263, *df* = 92) for the comparison between single manual-scorings and manual consensus scorings and ‐0.08 (*p* = 0.459, *df* = 92) for the comparison between auto-scoring and manual consensus scorings.

Even though all scorers participating in the original studies were highly trained and experienced technologists, there were substanatial differences between the lowest and highest kappa values between two experts (see Supplementary Figure S8). In Dataset B for instance Cohen’s kappa ranged between 0.461 (scorer 2 vs. scorer 5) and 0.785 (scorer 8 vs. scorer 9). This range was smaller when comparing the auto-scoring to the manual-scorings (0.615 comparing Somnolyzer against scorer 5 and 0.711 comparing Somnolyzer against scorer 9). The highest agreements were observed in all three datasets between scorers from the same sleep center, while the lowest agreements were between scorers from different centers.

## Discussion

We have demonstrated that (1) sleep staging ambiguity is the rule rather than the exception; (2) consequently, a hypnodensity chart representing sleep stage probabilities is more appropriate to visualize sleep architecture than a hypnogram; (3) sleep stage probabilities can be estimated almost perfectly by an auto-scoring system; (4) compared to manual consensus scoring, sleep staging derived from auto-scoring is for each individual PSG noninferior to manual-scoring; and finally (5) auto-scoring agrees with the scoring of at least one expert scorer for 98%–99% of epochs.

There is a common belief in the field that sleep stage disagreement across scorers is limited to 20%–30% of epochs [36], supported by studies reporting agreement for approximately 70%–80% of epochs [4, 5, 37]; however, studies comparing six or more scorers have questioned this conclusion [8]. In the current paper, we systematically assessed agreement across 2–12 scorers, and found that the observed decline in sleep staging agreement perfectly followed a power function with an exponent close to ‐0.5. Thus, scoring agreement is approximately inversely proportional to the square root of the number of scorers. In one dataset, on average only 32% of epochs were scored unequivocally by the 12 scorers. By extrapolating the power function to a larger number of scorers, our results support the hypothesis that the opposite of the common belief is true: 70%–80% of epochs will demonstrate some degree of sleep staging ambiguity across scorers. We observed that the exponent of each power function was very similar across datasets, supporting the robustness of this finding despite differing sample sizes, patient/participant characteristics, and the number and expertise of the scorers. The exponent was, however, dependent on sleep stage. While the agreement for epochs scored as N2 was similar to that observed over all stages, the agreement was higher for stages Wake and REM, lower for stage N3, and lowest for stage N1. Based on these models, we can expect sleep staging agreement of <50% for W or R if ≥16 scorers are compared; < 50% for N2 if ≥6 scorers are compared, <50% for N3 if ≥3 scorers are compared, and <50% for N1 if only ≥2 scorers are compared, again emphasizing that sleep stage ambiguity is the rule rather than the exception.

In 2016, Younes et al. published an analysis of inter-scorer variability in sleep staging by comparing six scorings from two experienced and highly trained technologists: two initial independent scorings, two scorings after editing their own initial scoring months later, and two scorings after editing the same auto-scoring [38]. The authors reported complete agreement between the six scorings in 66 ± 13% of all epochs. In their analysis, only few of the 34% epochs with disagreements were attributable to scorer error or scorer bias. Instead, the vast majority (82%) of the epochs with disagreements were due to a large and highly variable number of equivocal epochs ranging from 3% to 76% of all epochs for the 56 PSGs included in the study (mean 28 ± 13%). Thus, depending on PSG signal characteristics, in up to 76% of all epochs at least one of the scorers had revised their earlier assigned sleep stage or accepted a different sleep stage made by an auto-scoring algorithm. The authors concluded that digital identification of key staging variables such as sleep spindles, delta waves, or objective measures of sleep depth might significantly improve inter-scorer reliability. As described in the on-line supplement, we used such sleep/wake related neurological features as inputs to our artificial intelligence based sleep scoring algorithm to estimate sleep stage probabilities, thereby identifying equivocal epochs and the extent of their ambiguity. In the present datasets with multiple scorers from several scoring centers, we observed the highest agreement in all three datasets between scorers from the same center, while the lowest agreements were between scorers from different centers. This finding indicates that scoring bias between two technologists might be reduced by aligning and agreeing on the interpretation of the scoring guideline. Interestingly, however, the technologists with the highest agreement to each other were not necessarily those with the highest agreement to the consensus scoring. As shown in Supplementary Figure S8, the highest agreement in Dataset C was between scorers 10 and 12 (Cohen’s kappa 0.782), while the agreement between scorer 12 and the consensus scoring was low (Cohen’s kappa 0.642). Indeed, only scorer 11 showed a lower agreement with the consensus, while the remaining ten scorers showed a higher agreement with the consensus. Thus, high agreement between two scorers does not necessarily indicate increased validity of their scorings as compared to a consensus scoring derived from a larger group of experts. In fact, it could also mean that they have a similar scorer bias, possibly introduced by internal alignment on the interpretation of the scoring guideline. Both, manual- and auto-scoring, showed high agreements to the (unbiased) consensus scoring, typically higher than the agreements with individual scorings. This finding, once again, clearly demonstrates the value of having a gold standard based on multiple scorings. Last but not least, the presented auto-scoring system agrees better with the consensus scoring than the best of the manual scorers in all three datasets, supporting the quality and validity of the auto-scoring output.

It is clear that a hypnogram produced in clinical practice by a single technologist may be misleading, since it does not reflect sleep stage ambiguities. A hypnodensity chart, however, avoids this over-simplification by presenting sleep stage probabilities rather than definitive sleep stages. Such sleep stage probabilities can be determined based on manual-scoring if multiple scorers are available; however, this is not feasible in the clinical setting. As shown previously [23] and confirmed in the present study, a machine learning approach can produce sleep stage probabilities with a high degree of accuracy against different manual-scoring comparators, allowing for hypnodensity charts to be constructed in order for sleep stage ambiguity to be interpreted in clinical practice without the need for multiple scorers. The ICCs for absolute agreement between manual- and Somnolyzer-derived sleep stage probabilities showed excellent agreement (>0.90) for all datasets; indeed, even the lower-bound of the 95% confidence intervals exceed 0.9. Most importantly, we observed ICC values above 0.8 for the five-stage comparison for all individual PSGs, and above 0.5 for each individual sleep stage with only three exceptions resulting from <3.5 min of the relevant sleep stage.

The Somnolyzer auto-scoring algorithm applies a bi-directional long short-term memory (LSTM) recurrent neural network (RNN) to sleep/wake features as used during manual-scoring for staging sleep (input) to generate sleep stage probabilities per epoch (output); an approach which we have applied successfully to other scoring challenges such as sleep staging from cardio-respiratory features and detection of nocturnal scratching from actigraphy [39–42]. RNNs differ from feed-forward neural networks by redirecting outputs back to inputs. This enables the model to consider context from the past (as well as the future when using bi-directional RNNs), which makes it especially suitable for modeling spatio-temporal data. Thus, the assignment of a sleep stage to a given 30-second epoch considers not only the features derived from that epoch, but rather the feature distribution throughout the entire recording is considered.

The traditional metric for evaluating sleep staging agreement is Cohen’s kappa based on the hypnogram. When sleep stage probabilities were used to assign a single sleep stage to each epoch, auto-scoring demonstrated substantial agreement for the five-stage comparison against individual scorers (kappa 0.64–0.69) and the consensus of the scorers (kappa 0.75–0.78). In accordance with previous findings [23], the model accuracy vs. consensus scoring was always higher than the accuracy vs. individual scorers, indicating the value of multiple scorers for reliability studies. Note that Dataset A has been used previously be Stephansen et al.[23], thus our results can be directly compared. Stephansen et al. reported that their model achieved an average Cohen’s kappa value of 0.75 when compared to the unbiased consensus scorings, while our auto-scoring system achieved an average kappa value of 0.78.

Since all of our datasets included only expert scorers, it is reasonable to accept the sleep staging from any individual scorer as an acceptable interpretation of the neurological signals. Comparing Somnolyzer sleep staging against the sleep staging of any human scorer resulted in Cohen’s kappa values of 0.96–0.99 representing almost-perfect agreement. Across all sleep stage comparisons and regardless of the comparator adopted, the kappa values for Somnolyzer vs. manual-scoring were higher than those generated across manual scorers. Our study therefore shows the limitations of manual sleep staging on the one hand, and the opportunities offered by auto-scoring on the other. To our knowledge, this is the first study presenting noninferiority for each individual PSG. As shown in Figure 5, auto-scoring achieved greater agreement to the consensus than even the best of the scorers in 47% of the PSGs, and achieved greater agreement than at least one of the scorers in 100% of the PSGs. Consequently, we conclude that the Somnolyzer auto-scoring algorithm produces sleep staging that is ready for review and interpretation by a physician without the need for manual adjustment.

As expected, the kappa values vs. individual scorers and the unbiased consensus of the scorers were highest for stage W and R and lower for the NREM sub-classification, particularly for N1. Despite the large differences in kappa for the different sleep stages, all kappa values were close to 1 when compared to any scorer, independent of the sleep stage (Table 3). Thus, even for the most ambiguous stages N1 and N3, whenever auto-scoring assigned N1 or N3, almost always at least one scorer agreed with the auto-scoring (see also Supplementary Table S3).

When compared to the consensus scoring, the major confusion for epochs scored as N3 by the auto-scoring was with stage N2. However, the direction of these confusions were very different between the three test studies, resulting in a high sensitivity for N3 in Dataset A (92%) with a rather low precision (PPV of 60%), balanced sensitivity, and precision in the Dataset B (83% and 81%, respectively), and rather low sensitivity (66%) but high precision (92%) in Dataset C (Supplementary Table S2). Since all PSGs have been analyzed with the same auto-scoring system, most of this observed variability in sensitivity and precision must be due to the large inter-rater differences in scoring stage N3 sleep [8].

In recent years, several other investigators have applied neural network approaches to sleep staging with large differences in the size of the training data (from 80 PSGs scored by 5 scorers [43] to 19 924 PSGs [44]) and the testing approaches (internal testing including leave one subject out approach [45], leave one cohort out approach [46], *n*-fold cross-validation [43, 47–50], and holdout data [24, 27, 44, 46, 51] vs. external testing in datasets unseen by the model during training [7, 23–25, 27, 44, 52–54]). The reported kappa values ranged for the 14 studies with internal testing from 0.74 to 0.85 (mean kappa of the 14 studies with internal testing: 0.80 ± 0.03). In contrast, the kappa values for the 13 studies with external testing ranged from 0.60 to 0.80 (mean kappa of the 13 studies with external testing: 0.70 ± 0.06), where 0.60 was reported in a cohort of 77 patients with Parkinson’s disease [52] and 0.80 in a study of 25 healthy controls [54]. Consequently, performance of sleep scoring algorithms should be tested in datasets which are representative of the population to be tested and completely unseen by the model both during training and validation [46]. Note that we achieved here in the three datasets kappa values between 0.75 and 0.78 as compared to the consensus of multiple scorers, which is well within the range of kappa values reported for external testing. Thus, there is now convincing evidence that sleep staging using neural network approaches can achieve agreement with the “gold-standard” of manual-scoring with accuracy comparable to the inter-scorer reliability across scorers, suggesting that these artificial intelligence systems are valid alternatives to manual-scoring while having the advantages of efficiency and consistency.

Future studies should investigate whether the traditional assignment of a single sleep stage per epoch (hypnogram) might be replaced by sleep stage probabilities (hypnodensity) reflecting sleep stage ambiguity, given that all parameters required for reporting sleep scoring data per AASM guidance can be determined directly from sleep stage probabilities. For example, if the sleep stage probabilities are based on data from multiple scorers, the area under the probability curves per sleep stage (scaled in minutes) gives exactly the same values as obtained by averaging the time spent in each sleep stage across scorers. Since the sleep stage probabilities estimated by Somnolyzer demonstrated almost-perfect agreement with the manually derived sleep stage probabilities, the area under the algorithm-derived probability curves are also excellent estimate of the time in each sleep stage. Similarly, sleep stage probabilities can be used to generate all other commonly reported metrics such as sleep latency (the time between lights-off and the first epoch with a sleep probability >0.5) and REM latency (the time between sleep onset and the first epoch with a REM probability >0.5).

We also recommend that further head-to-head analyses are undertaken to evaluate the various publicly or commerciallyavailable sleep staging tools using a large dataset with multiple scorers, such as the AASM inter-scorer reliability program [37]. A single PSG containing 150 epochs was provided by the AASM for validation of the Stephansen et al. [23] auto-scoring algorithm; however, a dataset of this size is not sufficient to support validity claims for a scoring system intended for widespread clinical use. Furthermore, for systems offering full PSG analysis including arousals, respiratory events, and periodic leg movement detection in addition to sleep staging, evaluation of the scoring performance of all scored events would be of great value for sleep laboratories. Access to quality assurance programs such as those utilized for accreditation of technologists would allow for more thorough and systematic assessments of auto-scoring performance, enabling clinicians, and researchers to determine the best fit-for-purpose approach for their needs.

## Supplementary Material

zsac154_suppl_Supplementary_Material

## Preprint repositories

This manuscript is not preprinted or published prior to submitting to *Sleep.*
