# Supplementary material for: Scoring sleep with artificial intelligence enables quantification of sleep stage ambiguity: hypnodensity based on multiple expert scorers and auto-scoring
Source: Sleep. 2022 Jul 3;46(2):zsac154. doi: 10.1093/sleep/zsac154 (PMC9905781; doi:10.1093/sleep/zsac154)

Supplementary Material to “Scoring sleep with artificial intelligence enables quantification of sleep stage ambiguity: Hypnodensity based on multiple expert scorers and auto-scoring”

Jessie P. Bakker[1] *, Marco Ross[2] *, Andreas Cerny[2], Ray Vasko[1], Edmund Shaw, [1] Samuel Kuna[3,4], Ulysses J. Magalang[5], Naresh M. Punjabi[6], and Peter Anderer[2] +.

[1] Philips Sleep & Respiratory Care; Pittsburgh PA, USA;
[2] Philips Sleep & Respiratory Care; Vienna, Austria;
[3] Perelman School of Medicine, University of Pennsylvania, Philadelphia, PA, USA;
[4] Corporal Michael J. Crescenz Veterans Affairs Medical Center, Philadelphia, PA, USA;
[5] Division of Pulmonary, Critical Care, and Sleep Medicine, The Ohio State University Wexner Medical Center, Columbus, OH, USA;
[6] Division of Pulmonary, Critical Care, and Sleep Medicine, University of Miami, Miami FL, USA.

*Co-first authors

+Corresponding author. Peter Anderer, Sleep & Respiratory Care, Philips Austria GmbH, Kranichberggasse 4, 1120 Vienna, Austria. Email: peter.anderer@philips.com.

Algorithm Input Signals

The auto-scoring system used all recorded frontal, central and occipital EEG channels, left and right EOG channels, as well as the chin EMG channel for feature extraction. Before feature extraction, line interferences and its harmonics as well as ECG interferences were minimized and epochs with artifacts were identified. By default, the F4-M1 channel was used for slow-wave and k-complex detection, the O2-M1 channel for the detection of episodes with alpha waves, the C4-M1 channel for the determination of the EEG background activities (delta, theta, alpha, slow and fast beta activities) and both C4-M1 and C3-M2 channels for detection of sleep spindles. When artifact was present in these channels, the system automatically used the backup channels F3-M2, O1-M2 and C3-M2. Both EOG channels were used for detecting slow and rapid eye movements and the EOG channels together with the frontal EEG channels were used to detect eye-blinks. The chin EMG channel was used to detect tonic and transient EMG activities.

Classifier Architecture

The original Somnolyzer algorithm (Version 1.7; 2005) was developed according to R&K criteria,[1] and subsequently modified (Version 1.8; 2009) to comply with the AASM 2007 criteria[2]. As described in a sleep stage transition analysis,[2] there were almost no differences in sleep/wake or NREM/REM discriminations when the algorithm based on R&K criteria was compared against the AASM criteria; thus, these components of the algorithm were not modified, but additional training was undertaken for NREM subclassification. For the latest version of Somnolyzer released commercially in 2021 (Version 4.0), a supervised deep learning algorithm was trained, during which 472 PSGs from the SIESTA database were used for parameter optimization and the remaining 116 PSGs were used for early-stopping to prevent the model from overfitting. Each PSG was scored by two independent technologists and one consensus scorer chosen from a pool of 30 scorers to obtain R&K sleep stage probabilities as training targets. In a further step, arousals, sleep spindles, and k-complexes were added to the feature set and a convolutional neural network (CNN) followed by another bidirectional long short-term memory (LSTM) layer was trained using data from 72 PSGs scored according to AASM criteria, in order to sub-classify NREM sleep stages. The resulting bidirectional LSTM recurrent neural network (RNN) was integrated in the Somnolyzer sleep scoring system Version 4.0.

The model input includes 54 sleep/wake related neurological features per 30-second epoch and consists of six layers: first, a perceptron layer; then three stacked bidirectional layers of LSTM cells; and thereafter two more perceptron layers. The last layer applies a softmax activation to its six neurons corresponding to

the six R&K sleep stage probabilities. In a further step, the probabilities of the R&K NREM sleep stages S1 and S2 are converted by a CNN and LSTM to the probabilities for the AASM NREM sleep stages N1 and N2. While all R&K sleep stage probabilities (including W, R, Stage-3 [S3] and Stage-4 [S4]) serve as input for the N1/N2 classification, their values remain unchanged except for the probabilities for S3 and S4 being combined to the probability for N3. Concerning the change from S3 and S4 to N3, note that the increase of 9.5 min from S3+S4 to N3, as revealed in the sleep transition analysis, was completely explained by switching the detection of slow waves from the central to the frontal leads.[2] Thus, no adjustments other than detecting slow waves from frontal leads were necessary when adapting the scoring of deep sleep from R&K to AASM criteria.

The final network output assigned AASM-related sleep stage probabilities of Wake, N1, N2, N3, and REM to each 30-sec epoch.

Sleep stage was assigned to each epoch as follows: if the Wake-probability was > 0.5, assign W; else if the REM-probability was higher than the NREM probability, assign R; else if the N3-probability was higher than the sum of the N1- and N2-probabilities, assign N3; else if the N2-probability was higher than the N1-probability, assign N2; otherwise assign N1. An additional post-processing step was performed to enforce AASM smoothing rules for scoring R as well as N2 based on the occurrence (start and duration) of arousals, sleep spindles and K complexes.

SUPPLEMENTARY REFERENCES

1. Anderer P, Gruber G, Parapatics S, et al. An E-health solution for automatic sleep classification according to Rechtschaffen and Kales: validation study of the Somnolyzer 24 x 7 utilizing the Siesta database. *Neuropsychobiology*. 2005;51(3):115-33. doi:10.1159/000085205
2. Anderer P, Moreau A, Woertz M, et al. Computer-assisted sleep classification according to the standard of the American Academy of Sleep Medicine: validation study of the AASM version of the Somnolyzer 24 x 7. *Neuropsychobiology*. 2010;62(4):250-64. doi:10.1159/000320864

SUPPLEMENTARY FIGURE LEGENDS

Supplementary Figure S1: Percentage of epochs per sleep stage with complete agreement across all scorers in each dataset

The number of scorers compared is shown on the *x*-axis; the percentage of complete agreement across the compared scorers is shown on the *y*-axis. Within each panel depicting an individual sleep stage, the mean of all possible permutations for Dataset A is shown in yellow; Dataset B is shown in blue; Dataset C is shown in green (observed values; filled markers). The reduction in agreement alongside the increasing number of scorers follows an almost-perfect power function $y=ax^b$ (dashed lines for each dataset). Modelled together, the exponents for each power function are as follows: Stage Wake (W), b_{MEAN} = -0.23; Stage N1, b_{MEAN} = -2.05; Stage N2, b_{MEAN} = -0.47; Stage N3, b_{MEAN} = -0.95; Stage REM (R), b_{MEAN} = -0.26. Note, the decline in agreement with increasing numbers of manual scorers was for epochs scored as N2 similar to the decline observed as for all stages. For epochs scored as W and R the decline was weaker indicating lower disagreement between scorers for stages W and R, while for epochs scored as N3 and N1 the decline was stronger indicating higher disagreement between scorers for stages N3 and specifically N1.

Supplementary Figure S2: Hypnograms and hypnodensity charts derived from manual scoring for PSG-73

The PSG depicted here (from Dataset B) contains 0.5 minutes of N3 averaged across the nine scorers. Individual hypnograms and the hypnodensity chart, based on sleep stage probabilities derived from the scorers are shown. Color codes for the hypnodensity chart are: Wake [W]: gray; N1: cyan; N2: blue; N3: green; REM [R]: red.

Supplementary Figure S3: Hypnodensity charts derived from manual- and auto-scoring for PSG-73

The PSG depicted here is the same as the PSG in Supplementary Figure S2, containing 0.5 minutes of N3 averaged across the nine scorers. Sleep stage probabilities for individual sleep stages (Panels 1-5) derived from the scorers (left) and Somnolyzer auto-scoring (right). The probability distributions were compared quantitatively with an ICC value as shown. Panel 6 shows the hypnodensity charts that combine the individual sleep stage probabilities into a stacked area graph for manual- (left) and auto-scoring (right), also compared quantitatively with an ICC value. Color codes for the hypnodensity chart are shown in the legend (Wake [W]: gray; N1: cyan; N2: blue; N3: green; REM [R]: red). Finally, Panel 7 shows a hypnogram derived from majority vote of manual- (left) and auto-scoring (right), which can be compared quantitatively with a Cohen's kappa value as shown.

Supplementary Figure S4: Hypnograms and hypnodensity charts derived from manual scoring for PSG-81

The PSG depicted here (from Dataset B) contains 1.5 minutes of R averaged across the nine scorers. See Supplementary Figure S2 for an explanation.

Supplementary Figure S5: Hypnodensity charts derived from manual- and auto-scoring for PSG-81

The PSG depicted here is the same as the PSG in Supplementary Figure S4, containing 1.5 minutes of R averaged across the nine scorers. See Supplementary Figure S3 for an explanation.

Supplementary Figure S6: Hypnograms and hypnodensity charts derived from manual scoring for PSG-89

The PSG depicted here (from Dataset C) contains 3.5 minutes of N3 averaged across the twelve scorers. See Supplementary Figure S2 for an explanation.

Supplementary Figure S7: Hypnodensity charts derived from manual- and auto-scoring for PSG-89

The PSG depicted here is the same as the PSG in Supplementary Figure S6, containing 3.5 minutes of N3 averaged across the twelve scorers. See Supplementary Figure S3 for an explanation.

Supplementary Figure S8: Agreement between sleep stage assignments derived from manual- and auto-scoring for each individual scorer

Cohen's kappa values for manual- and auto-scored sleep staging vs. each other manual scoring as well as vs. the unbiased consensus (majority vote) of the remaining scorers for all three datasets. The line markers represent the kappa values between each individual scorer and all other scorers of the same dataset, the square markers represent the kappa values to the the consensus.

Note the large variability of pair-wise agreements (lines) between manual scorers (dataset A: lowest kappa value 0.517 between scorer 1 and 5 from two different sleep centers and highest kappa value 0.734 between scorers 3 and 4 from the same sleep center; dataset B: lowest kappa value 0.461 between scorer 2 and 5 from two different sleep centers and highest kappa value 0.785 between scorers 8 and 9 from the same sleep center; dataset C: lowest kappa value 0.486 between scorer 5 and 11 from two different sleep centers and highest kappa value 0.728 between scorers 10 and 12 from the same sleep center). Note that auto-scoring consistently showed higher agreement with the consensus than either of the manual scorers.

Supplementary Table S1: Confusion matrix for epoch-by-epoch comparisons of auto-scoring against individual scorers

		**Comparator 1: Individual scorers** Overall agreement between auto-scoring and comparator: Dataset A 78.4%, Dataset B 76.1%, Dataset C 74.2%														
		**W**			**N1**			**N2**			**N3**			**R**		
		**Dataset A**	**Dataset B**	**Dataset C**	**Dataset A**	**Dataset B**	**Dataset C**	**Dataset A**	**Dataset B**	**Dataset C**	**Dataset A**	**Dataset B**	**Dataset C**	**Dataset A**	**Dataset B**	**Dataset C**
**Somnolyzer Auto-scoring**	**W**	51989	13751	20362	4939	1834	1991	2982	770	555	114	44	12	921	287	168
		85.7%	81.1%	75.6%	15.1%	11.1%	11.0%	1.5%	1.4%	1.2%	0.3%	0.4%	0.2%	1.4%	2.2%	1.2%
	**N1**	5054	2498	4431	15865	9403	9821	14060	7159	6217	130	66	28	1748	953	495
		8.3%	14.8%	16.4%	48.5%	56.5%	54.4%	7.0%	13.3%	13.6%	0.3%	0.5%	0.3%	2.7%	7.4%	3.6%
	**N2**	2190	454	1796	6453	4180	5639	156453	42245	36905	11631	2807	3909	1980	570	806
		3.6%	2.7%	6.7%	19.7%	25.1%	31.3%	78.1%	78.3%	80.9%	29.6%	23.7%	51.0%	3.1%	4.4%	5.8%
	**N3**	193	5	12	37	19	12	19699	2819	1384	27323	8936	3716	1	2	0
		0.3%	0.1%	0.0%	0.1%	0.1%	0.1%	9.8%	5.2%	3.0%	69.5%	75.4%	48.5%	0.0%	0.0%	0.0%
	**R**	1229	241	328	5413	1180	592	7138	978	542	94	0	1	60127	11065	12336
		2.0%	1.4%	1.2%	16.5%	7.1%	3.3%	3.6%	1.8%	1.2%	0.2%	0.0%	0.0%	92.8%	85.9%	89.4%
	**PPV**	85.3%	82.4%	88.2%	43.0%	46.8%	46.8%	87.5%	84.1%	75.2%	57.8%	75.9%	72.6%	81.2%	82.2%	89.5%

Data are presented as the number of epochs with agreement between auto-scoring and the comparator, above the percentage of comparator epochs (sensitivity values). The final row presents positive predictive values (PPV), also referred to as precision values.

The comparator is the individual scorer, which is a pairwise comparison between the evaluated scorer (manual or auto-scoring) and each remaining scorer. Note that the use of a different comparator in Supplementary Tables 1-3 results in a different number of epochs for which agreement is evaluated.

Supplementary Table S2: Confusion matrix for epoch-by-epoch comparisons of auto-scoring against unbiased consensus of the scorers

		**Comparator 2: Unbiased consensus of scorers**<br>Overall agreement between auto-scoring and comparator: Dataset A 85.4% Dataset B 83.1%; Dataset C 83.2%														
		**W**			**N1**			**N2**			**N3**			**R**		
		**Dataset A**	**Dataset B**	**Dataset C**	**Dataset A**	**Dataset B**	**Dataset C**	**Dataset A**	**Dataset B**	**Dataset C**	**Dataset A**	**Dataset B**	**Dataset C**	**Dataset A**	**Dataset B**	**Dataset C**
**Somnolyzer Auto-scoring**	**W**	9216	1625	1778	685	136	99	205	71	30	8	3	0	70	19	8
		91.3%	87.4%	80.7%	13.2%	9.0%	8.0%	0.6%	1.1%	0.7%	0.2%	0.2%	0.0%	0.6%	1.3%	0.7%
	**N1**	625	206	331	3269	1114	909	2096	822	475	2	0	0	174	89	35
		6.2%	11.1%	14.9%	63.0%	73.5%	73.5%	6.0%	12.8%	11.6%	0.0%	0.0%	0.0%	1.5%	6.0%	2.9%
	**N2**	125	24	93	616	185	211	28633	3219	3531	380	127	202	94	29	51
		1.2%	1.3%	4.2%	11.9%	12.2%	17.1%	82.2%	81.3%	86.3%	7.5%	10.6%	33.9%	0.8%	2.0%	4.2%
	**N3**	24	1	1	1	0	0	3163	236	33	4695	1072	393	0	0	0
		0.2%	0.1%	0.0%	0.0%	0.0%	0.0%	9.1%	3.7%	0.8%	92.3%	89.2%	66.1%	0.0%	0.0%	0.0%
	**R**	99	4	3	618	80	17	745	73	21	0	0	0	10921	1339	1109
		1.0%	0.2%	0.1%	11.9%	5.3%	1.4%	2.1%	1.1%	0.5%	0.0%	0.0%	0.0%	97.0%	90.7%	92.2%
	**PPV**	90.5%	87.6%	92.9%	53.0%	49.9%	51.9%	95.9%	93.5%	86.4%	59.6%	81.0%	92.0%	88.2%	89.5%	96.4%

Data are presented as the number of epochs with agreement between auto-scoring and the comparator, above the percentage of comparator epochs (sensitivity values). The final row presents positive predictive values (PPV), also referred to as precision values.

The comparator is the unbiased consensus scoring, in which each evaluated scorer is compared to the consensus of the remaining scorers, and the auto-scoring is compared to the same unbiased consensus for each scorer. Note that the use of a different comparator in Supplementary Tables 1-3 results in a different number of epochs for which agreement is evaluated.

Supplementary Table S3: Confusion matrix for epoch-by-epoch comparisons of auto-scoring against any scorer

		Comparator 3: Any scorer<br>Overall agreement between auto-scoring and comparator: Dataset A 97.9%; Dataset B 98.3%; Dataset C 99.1%														
		**W**			**N1**			**N2**			**N3**			**R**		
		**Dataset A**	**Dataset B**	**Dataset C**	**Dataset A**	**Dataset B**	**Dataset C**	**Dataset A**	**Dataset B**	**Dataset C**	**Dataset A**	**Dataset B**	**Dataset C**	**Dataset A**	**Dataset B**	**Dataset C**
**Somnolyzer Auto-scoring**	**W**	9884	1812	1911	187	13	6	91	23	8	3	1	0	22	5	0
		97.9%	98.7%	98.5%	3.3%	0.6%	0.3%	0.3%	1.1%	0.2%	0.0%	0.0%	0.0%	0.2%	2.2%	0.0%
	**N1**	145	20	24	5355	2115	1699	632	62	24	2	0	0	34	34	3
		1.4%	1.1%	1.2%	95.2%	98.9%	98.9%	2.0%	1.1%	0.6%	0.0%	0.0%	0.0%	0.3%	2.2%	0.3%
	**N2**	37	4	6	35	4	12	29941	5558	4068	9	5	0	4	13	2
		0.4%	0.2%	0.3%	0.6%	0.2%	0.7%	97.3%	98.2%	99.2%	0.1%	0.4%	0.0%	0.0%	0.9%	0.2%
	**N3**	16	0	0	0	0	0	22	0	0	7716	1309	427	0	0	0
		0.2%	0.0%	0.0%	0.0%	0.0%	0.0%	0.1%	0.0%	0.0%	99.8%	99.5%	100%	0.0%	0.0%	0.0%
	**R**	11	0	0	48	7	1	97	16	0	0	0	0	12227	1473	1149
		0.1%	0.0%	0.0%	0.9%	0.3%	0.1%	0.3%	0.3%	0.0%	0.0%	0.0%	0.0%	99.5%	96.6%	99.6%
	**PPV**	97.0%	97.7%	99.3%	86.9%	94.8%	97.1%	99.7%	99.5%	99.5%	99.3%	100%	100%	98.7%	98.5%	99.9%

Data are presented as the number of epochs with agreement between auto-scoring and the comparator, above the percentage of comparator epochs (sensitivity values). The final row presents positive predictive values (PPV), also referred to as precision values.

The comparator is any scorer, in which each evaluated scorer is compared to all remaining scorers, where each epoch is considered correct if at least one of the remaining scorers agreed with the evaluated scorer, and the auto-scoring is compared to the same combinations of scorers. Note that the use of a different comparator in Supplementary Tables 1-3 results in a different number of epochs for which agreement is evaluated.

Supplementary Figure S1

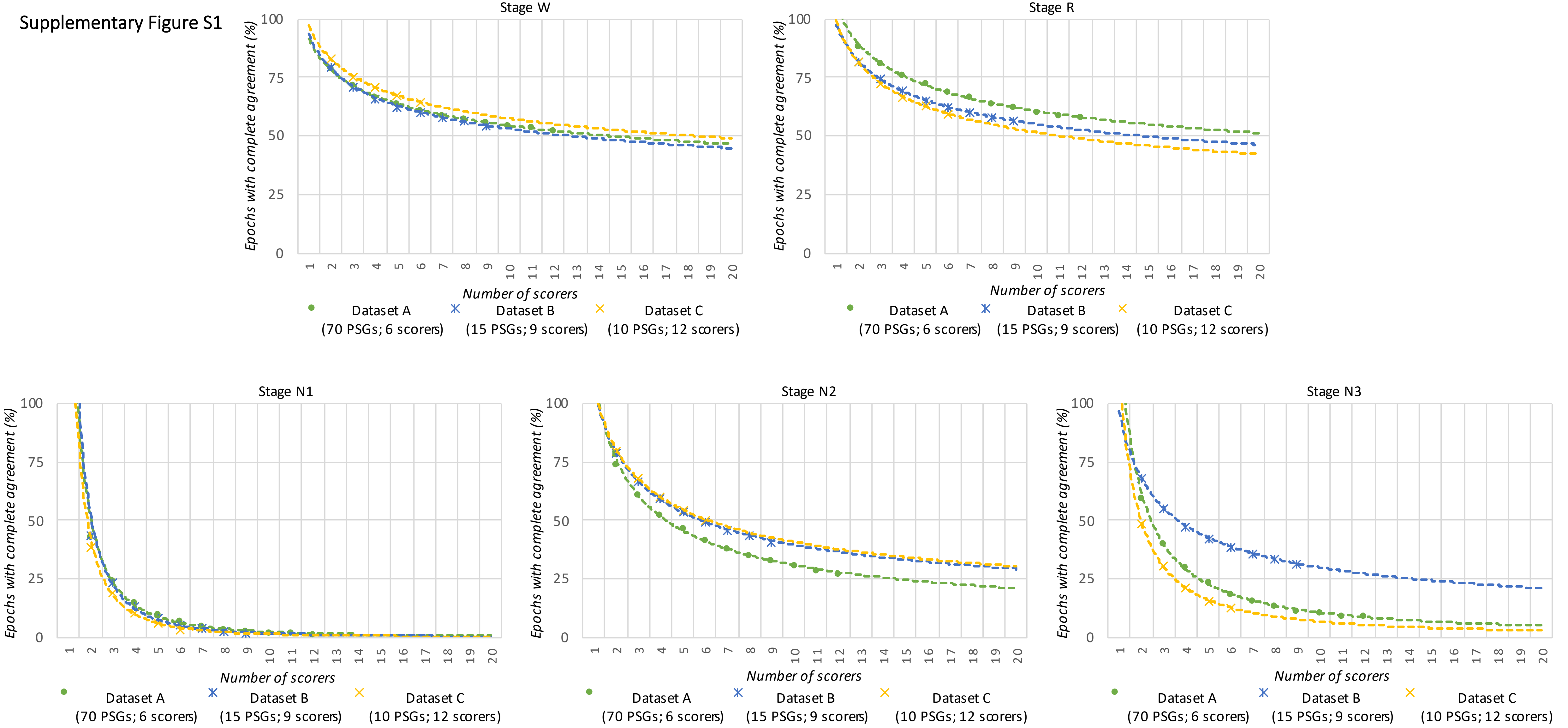

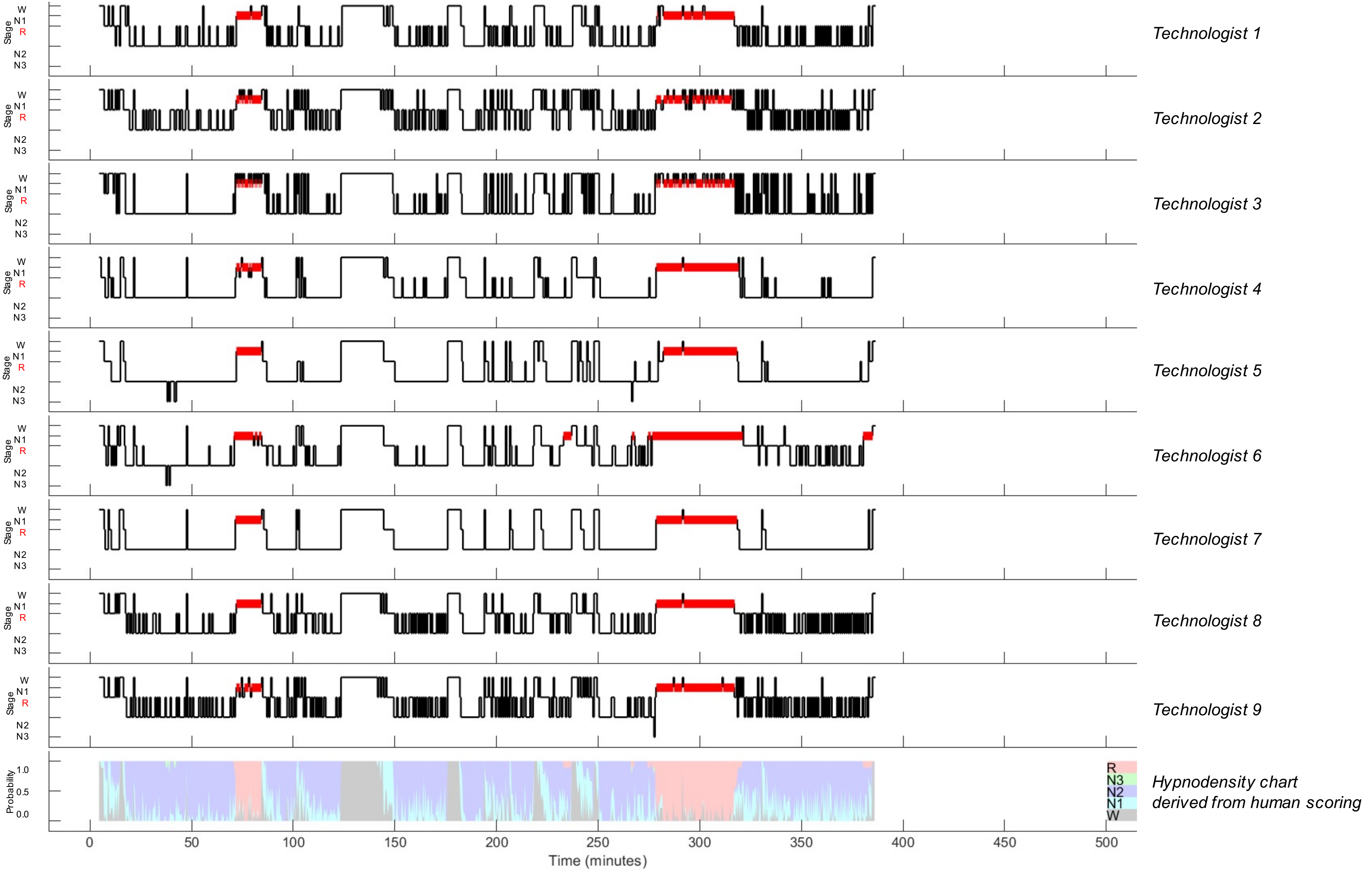

Supplementary Figure S2

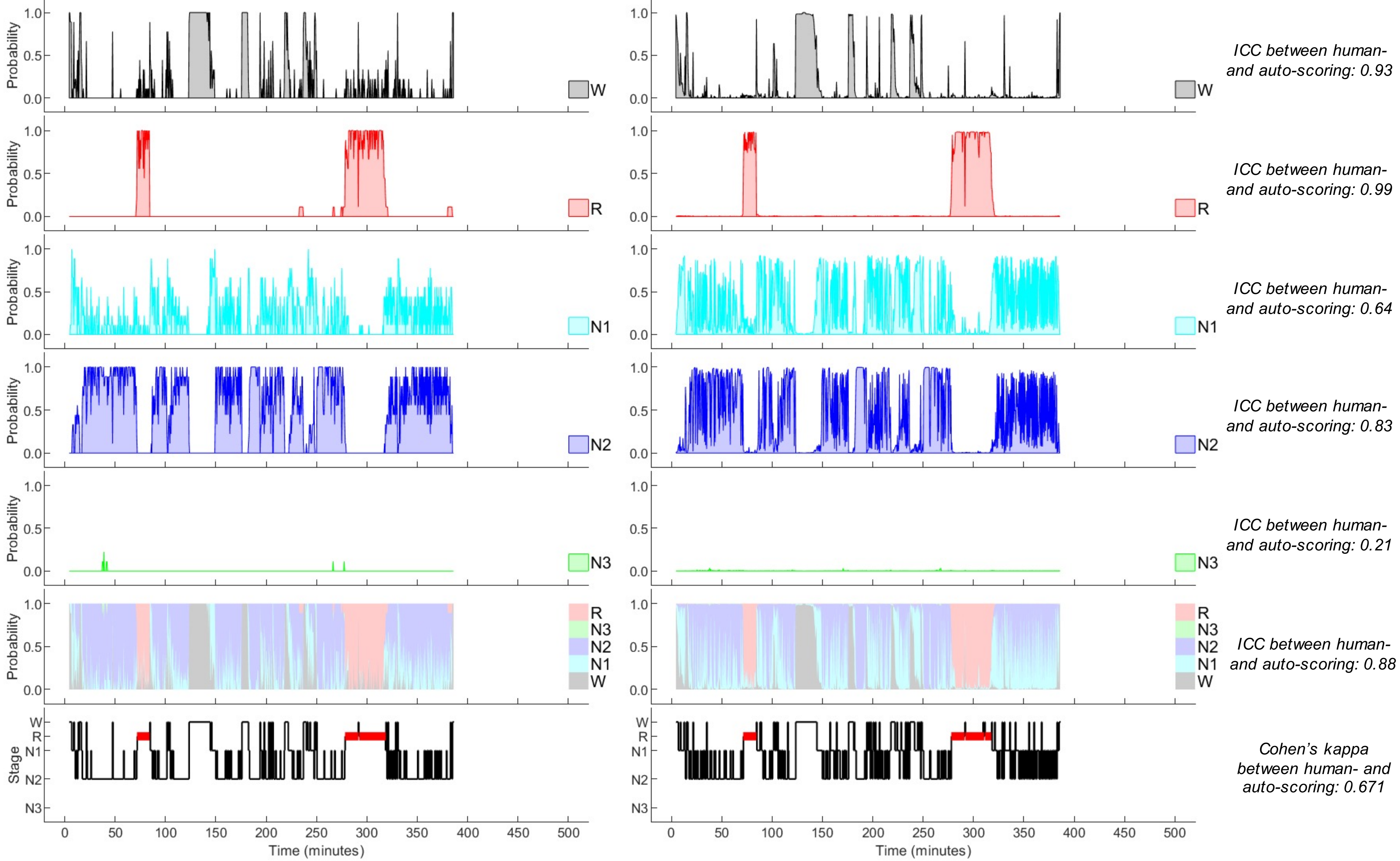

Supplementary Figure S3

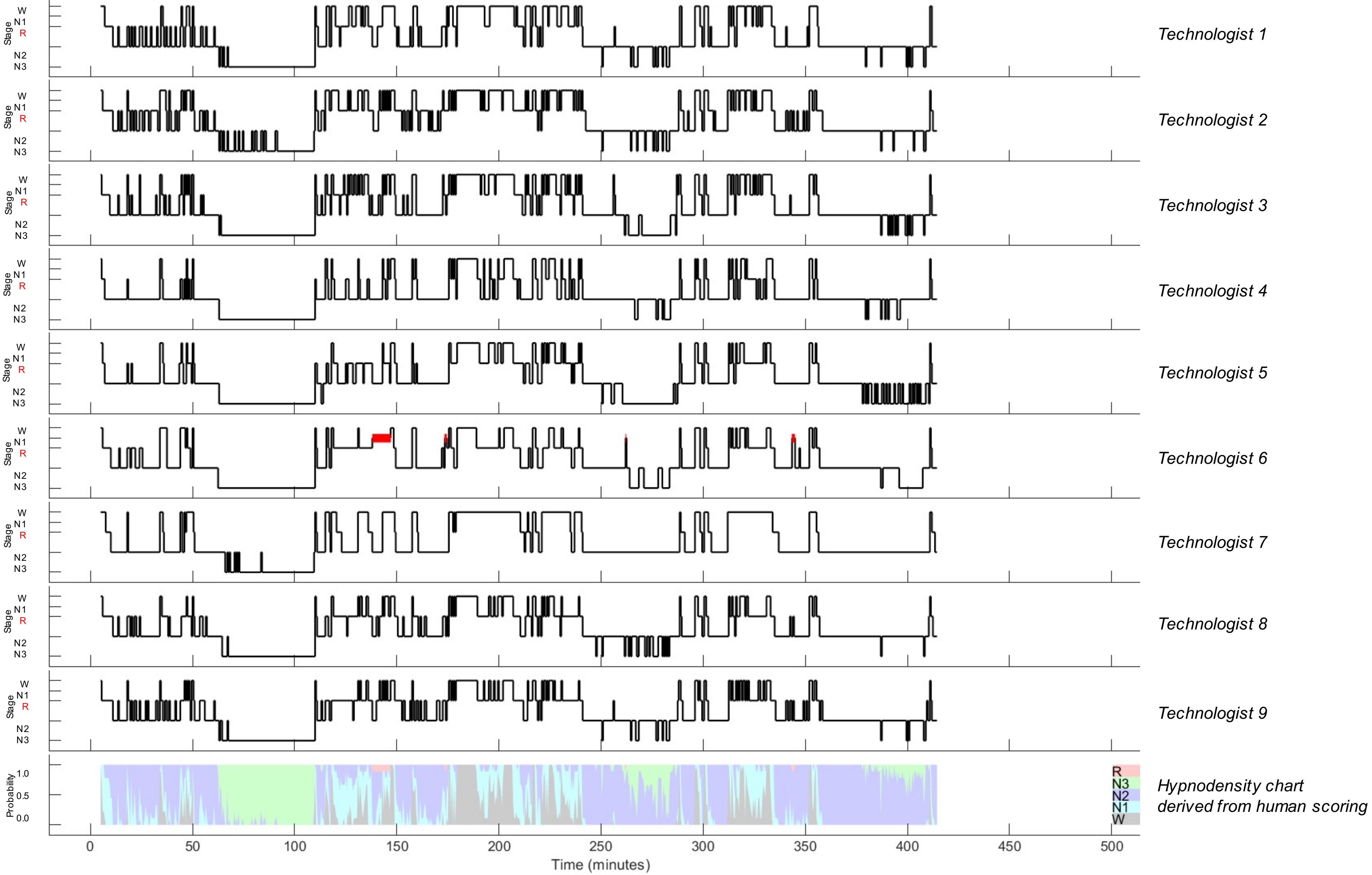

Supplementary Figure S4

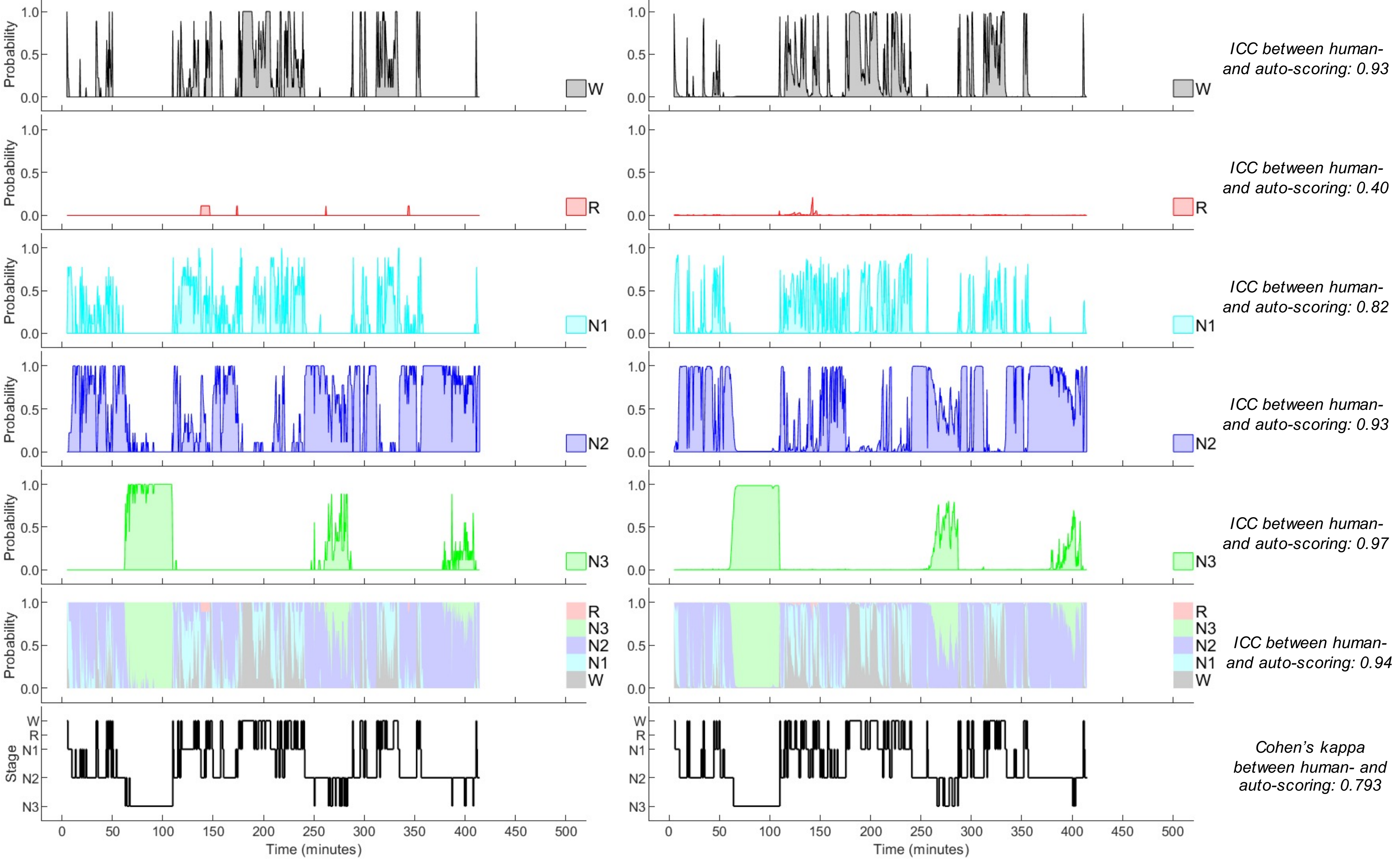

Supplementary Figure S5

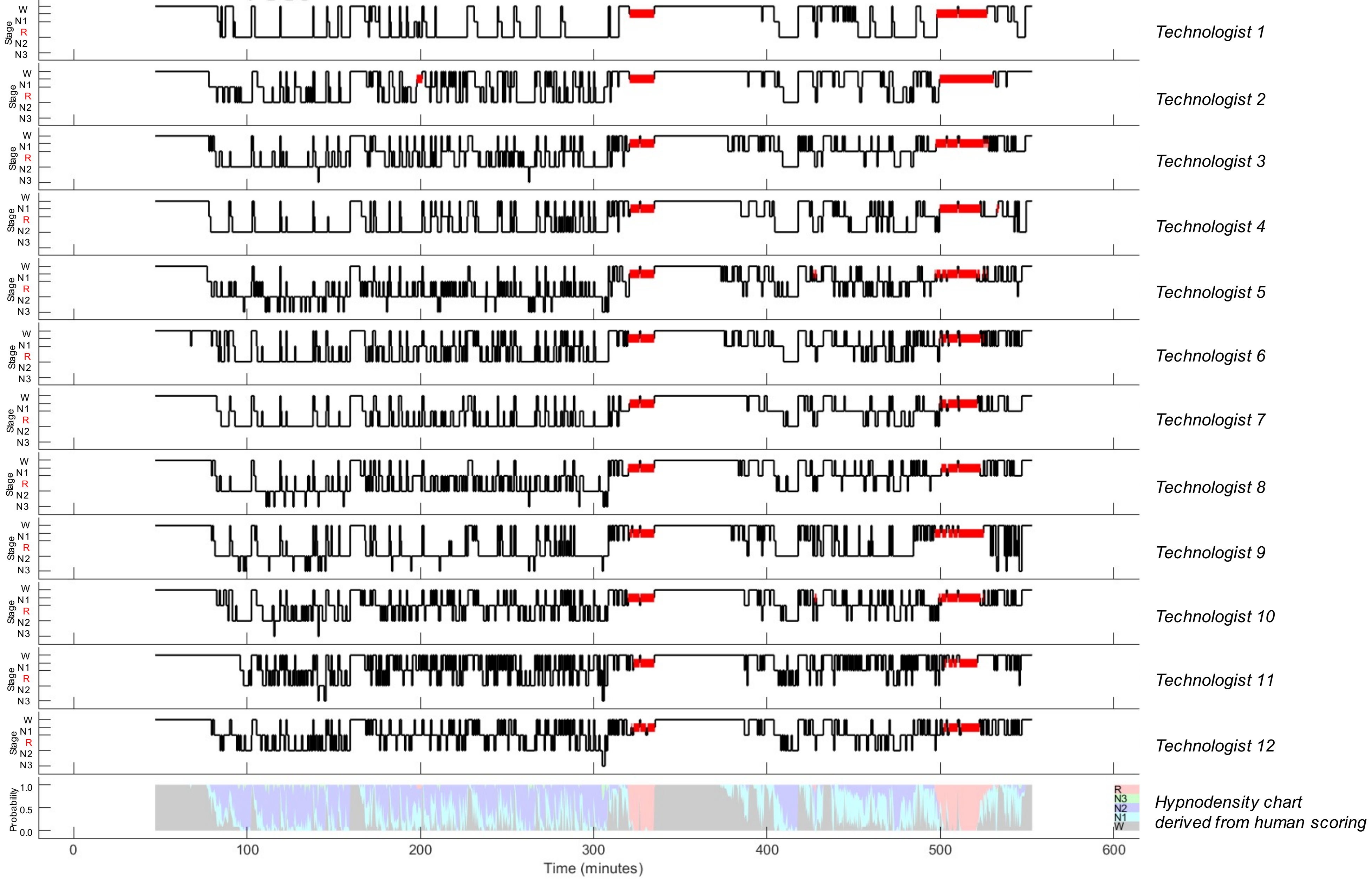

Supplementary Figure S6

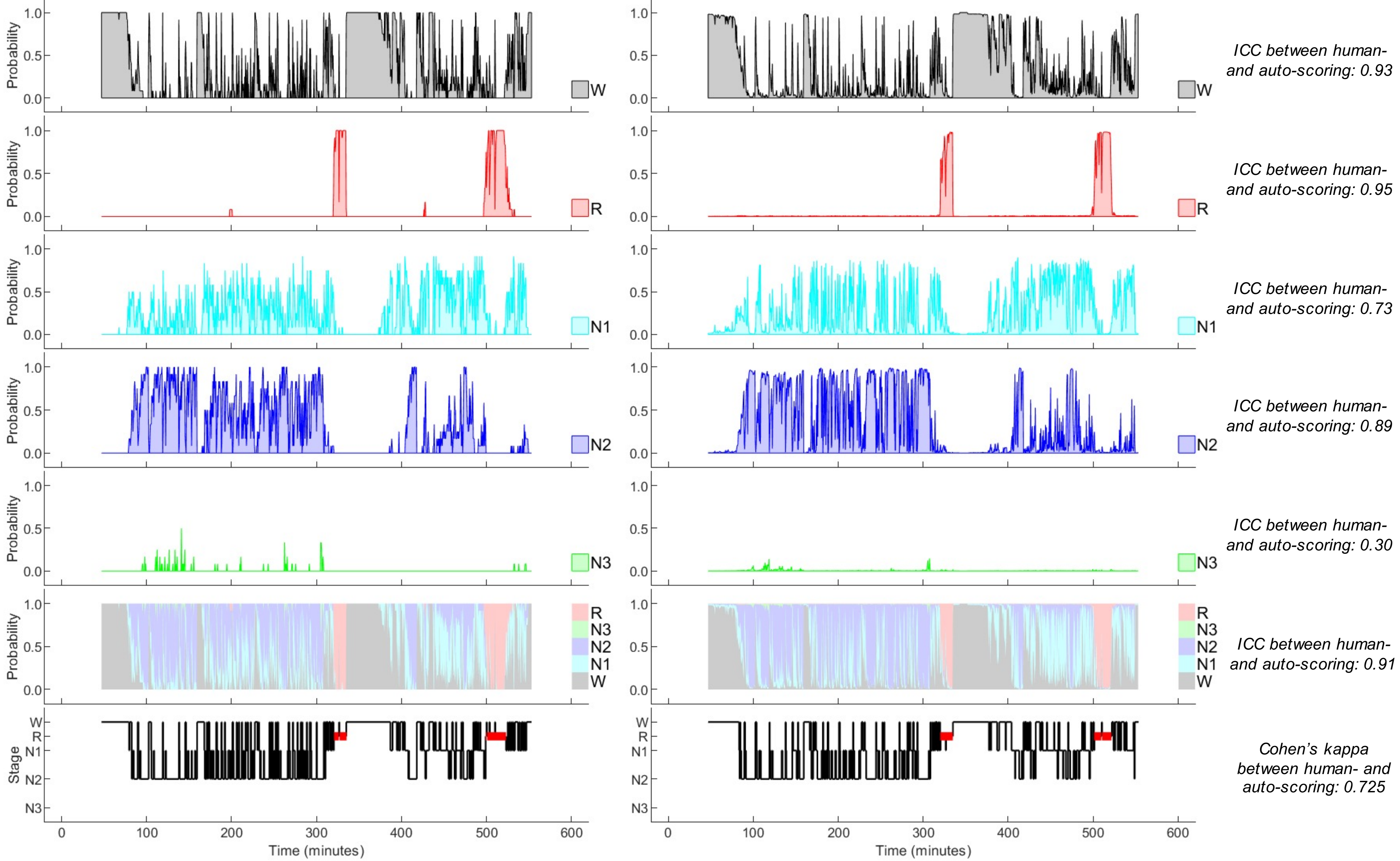

Supplementary Figure S7

Supplementary Figure S8

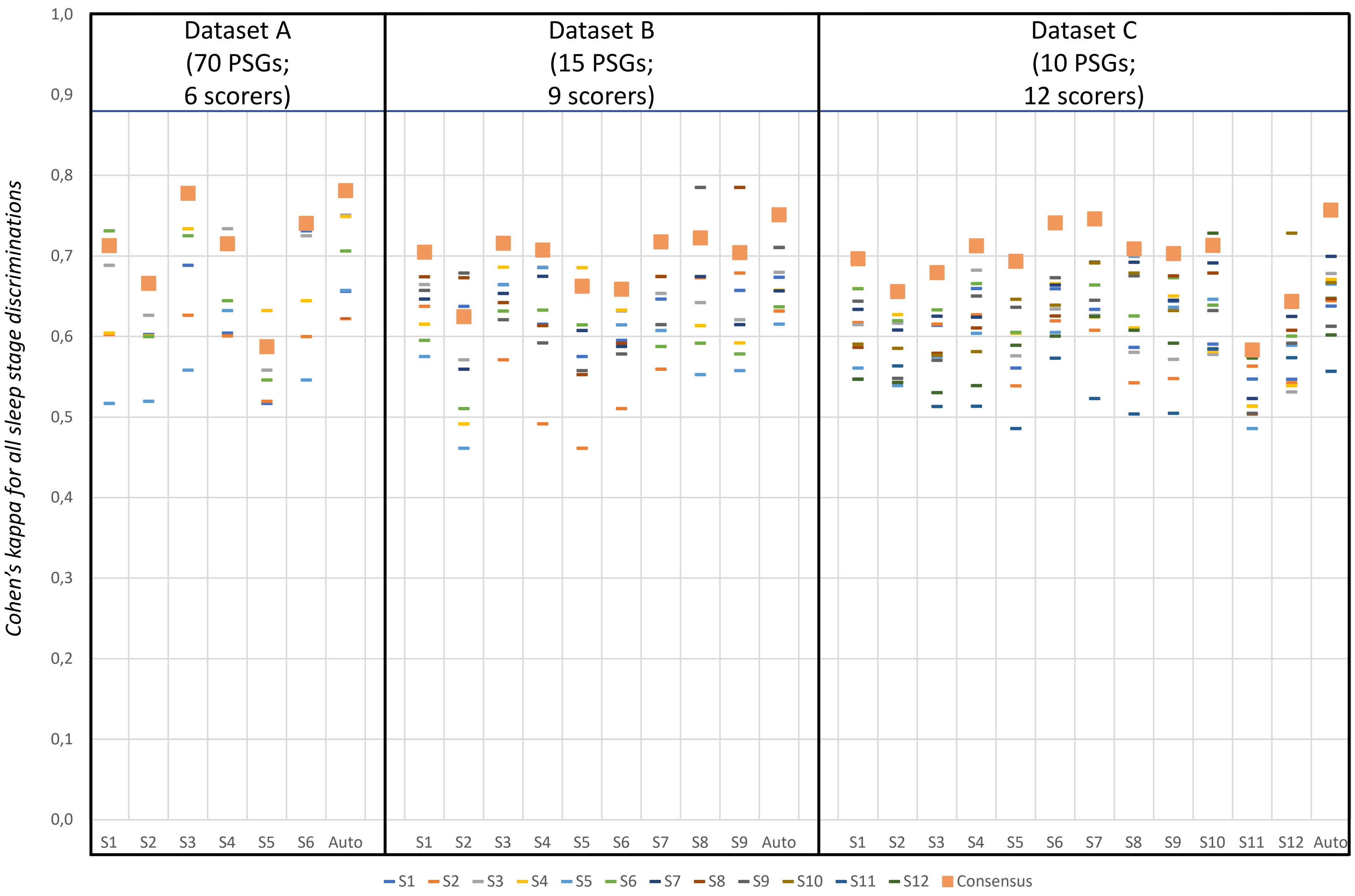

Supplement: zsac154_suppl_Supplementary_Material [file zsac154_suppl_supplementary_material.pdf]
